# Supplementary material for: Characteristics and interpretation of subgroup analyses based on tumour characteristics in randomised trials testing target-specific anticancer drugs: design of a systematic survey
Source: BMJ Open. 2020 May 30;10(5):e034565. doi: 10.1136/bmjopen-2019-034565 (PMC7264639; doi:10.1136/bmjopen-2019-034565)
Supplement: Supplementary data [file bmjopen-2019-034565supp001.pdf]

Supplement

PubMed search strategy:

("Lancet Haematol"[Journal] OR "J Clin Oncol"[Journal] OR "Ann Oncol"[Journal] OR "JAMA Oncol"[Journal] OR "Br J Cancer"[Journal] OR "Lancet Oncol"[Journal] OR "Cancer"[Journal] OR "Blood"[Journal] OR "Leukemia"[Journal]) OR (("N Engl J Med"[Journal] OR "Lancet"[Journal] OR "JAMA"[Journal] OR "Ann Intern Med"[Journal]) AND ("neoplasms"[MeSH Terms] OR cancer [sb] OR neoplas*[tiab] OR cancer*[tiab] OR malignan* OR tumor[tiab] OR tumour[tiab])) AND (randomized controlled trial[pt] OR controlled clinical trial[pt] OR randomized[tiab] OR placebo[tiab] OR "clinical trials as topic"[MeSH Terms:noexp] OR randomly[tiab] OR trial[ti] NOT ("animals"[MeSH Terms] NOT "humans"[MeSH Terms])) AND ("2014/01/01"[PDAT] : "3000/12/31"[PDAT])

Last date of search 20/09/2017; number of identified articles: 7729
