# Supplementary material for: Characteristics and interpretation of subgroup analyses based on tumour characteristics in randomised trials testing target-specific anticancer drugs: design of a systematic survey
Source: BMJ Open. 2020 May 30;10(5):e034565. doi: 10.1136/bmjopen-2019-034565 (PMC7264639; doi:10.1136/bmjopen-2019-034565)
Supplement: Author's manuscript [file bmjopen-2019-034565.draft_revisions.pdf]

BMJ Open

BMJ Open is committed to open peer review. As part of this commitment we make the peer review history of every article we publish publicly available.

When an article is published we post the peer reviewers' comments and the authors' responses online. We also post the versions of the paper that were used during peer review. These are the versions that the peer review comments apply to.

The versions of the paper that follow are the versions that were submitted during the peer review process. They are not the versions of record or the final published versions. They should not be cited or distributed as the published version of this manuscript.

BMJ Open is an open access journal and the full, final, typeset and author-corrected version of record of the manuscript is available on our site with no access controls, subscription charges or pay-per-view fees (http://bmjopen.bmj.com).

If you have any questions on BMJ Open's open peer review process please email info.bmjopen@bmj.com

Characteristics and interpretation of subgroup analyses based on molecular alterations in randomized trials testing target-specific anti-cancer therapy: design of a systematic survey

Journal:	*BMJ Open*
Manuscript ID	bmjopen-2019-034565
Article Type:	Protocol
Date Submitted by the Author:	07-Oct-2019

Complete List of Authors:	Schandelmaier, Stefan; University of Basel, Institute for Clinical Epidemiology and Biostatistics, Department of Clinical Research; McMaster University, Health Research Methods, Evidence, and Impact<br>Schmitt, Andreas; University Hospital Basel, Department of Medical Oncology<br>Herbrand, Amanda; University Hospital Basel, Department of Medical Oncology<br>Glinz, Dominik; University of Basel, Institute for Clinical Epidemiology and Biostatistics, Department of Clinical Research<br>Ewald, Hannah; University of Basel, University Medical Library<br>Briel, Matthias; University of Basel, Institute for Clinical Epidemiology and Biostatistics, Department of Clinical Research; McMaster University, Health Research Methods, Evidence, and Impact<br>Guyatt, Gordon; McMaster University, Health Research Methods, Evidence, and Impact; McMaster University, Department of Medicine<br>Hemkens, Lars; University of Basel, Institute for Clinical Epidemiology and Biostatistics, Department of Clinical Research<br>Kasenda, Benjamin; University of Basel, Institute for Clinical Epidemiology and Biostatistics, Department of Clinical Research; Stuttgart Cancer Center / Cancer Centre Eva Mayr-Stihl

Keywords:	Precision medicine, Randomized controlled trials, Subgroup analysis, Systematic survey of studies, ONCOLOGY, STATISTICS & RESEARCH METHODS

SCHOLARONE™
Manuscripts

I, the Submitting Author has the right to grant and does grant on behalf of all authors of the Work (as defined in the below author licence), an exclusive licence and/or a non-exclusive licence for contributions from authors who are: i) UK Crown employees; ii) where BMJ has agreed a CC-BY licence shall apply, and/or iii) in accordance with the terms applicable for US Federal Government officers or employees acting as part of their official duties; on a worldwide, perpetual, irrevocable, royalty-free basis to BMJ Publishing Group Ltd ("BMJ") its licensees and where the relevant Journal is co-owned by BMJ to the co-owners of the Journal, to publish the Work in this journal and any other BMJ products and to exploit all rights, as set out in our licence.

The Submitting Author accepts and understands that any supply made under these terms is made by BMJ to the Submitting Author unless you are acting as an employee on behalf of your employer or a postgraduate student of an affiliated institution which is paying any applicable article publishing charge ("APC") for Open Access articles. Where the Submitting Author wishes to make the Work available on an Open Access basis (and intends to pay the relevant APC), the terms of reuse of such Open Access shall be governed by a Creative Commons licence – details of these licences and which Creative Commons licence will apply to this Work are set out in our licence referred to above.

Other than as permitted in any relevant BMJ Author's Self Archiving Policies, I confirm this Work has not been accepted for publication elsewhere, is not being considered for publication elsewhere and does not duplicate material already published. I confirm all authors consent to publication of this Work and authorise the granting of this licence.

For peer review only

Characteristics and interpretation of subgroup analyses based on molecular alterations in randomized trials testing target-specific anti-cancer therapy: design of a systematic survey

Authors

Stefan Schandelmaier (1,2), corresponding author:

Stefan Schandelmaier, MD PHD
Institute for Clinical Epidemiology and Biostatistics,
Department of Clinical Research,
University Hospital and University of Basel
Spitalstrasse 12, 4056 Basel, Switzerland
s.schandelmaier@gmail.com
+41 176 567 382 89

Andreas M Schmitt (3)

Amanda K Herbrand (3)

Dominik Glinz (1)

Hannah Ewald (4)

Matthias Briel (1,2)

Gordon Guyatt (2,5)

Lars G Hemkens (1)

Benjamin Kasenda (3,6)

Affiliations

(1) Institute for Clinical Epidemiology and Biostatistics, Department of Clinical Research, University Hospital and University of Basel, Switzerland
(2) Department of Health Research Methods, Evidence, and Impact, McMaster University, Hamilton, Canada
(3) Department of Medical Oncology, University Hospital Basel, Switzerland
(4) University Medical Library, University of Basel, Basel, Switzerland
(5) Department of Medicine, McMaster University, Hamilton, Canada
(6) Stuttgart Cancer Center / Cancer Centre Eva Mayr-Stihl, Department for Haematology/Oncology and

For peer review only - http://bmjopen.bmj.com/site/about/guidelines.xhtml

Palliative Care, Stuttgart, Germany

Word count abstract: 296

Word count main text: 2709

Abstract

Introduction: Target-specific anti-cancer drugs are under rapid development. Little is known, however, about the risk of administering target-specific drugs to patients whose tumors might carry molecular alterations that make the drug ineffective or even harmful. Possible interactions between target-specific anti-cancer drugs and molecular alterations are increasingly tested through subgroup analyses in randomized clinical trials (RCTs). Such subgroup analyses have the potential to be more credible and influential than subgroup analyses based on traditional factors such as sex or tumor stage. In addition, they may more frequently lead to qualitative subgroup effects, i.e., show benefit in one but harm in another subgroup of patients (e.g. if the molecular alteration makes the drug ineffective or even enhance tumor growth). If so, subgroup analyses based on molecular alterations would be highly relevant for patient safety. To systematically assess the frequency and characteristics of subgroup analyses based on molecular alterations, the frequency of qualitative subgroup effects, their credibility, and the interpretations that investigators and guidelines developers report.

Methods and analysis: We will perform a systematic survey of 433 randomized clinical trials (RCTs) testing the effect of target-specific anti-cancer drugs. Teams of methodologically trained investigators and oncologists will identify eligible studies, extract relevant data, and assess the credibility of putative subgroup effects using a recently developed formal instrument. Using qualitative and quantitative methods, we will systematically assess how trial investigators interpret apparent subgroup effects based on molecular alterations and the extent to which they influence subsequent practice guidelines. Our results will provide empirical data characterizing an increasingly used type of subgroup analyses in cancer trials and its potential impact on precision medicine to predict benefit or harm.

Ethis and dissemination: Formal ethical approval is not required for this study. We will disseminate the findings in a peer-reviewed and open-access journal publication.

For peer review only - http://bmjopen.bmj.com/site/about/guidelines.xhtml

Keywords

Oncology
Target-specific anti-cancer treatment
Precision medicine
Randomized controlled trials
Effect modification
Subgroup analysis
Reporting of research
Interpretation of research
Meta-research
Systematic survey of studies

List of abbreviations

BRAF = A proto-oncogene
EGFR = Epithelial Growth Factor Receptor
MA = Molecular alteration
RAS = Family of proteins relevant for intracellular signaling
RCT = randomized clinical trial

Strengths and limitations of this study

- This study will provide an systematic overview of subgroup analyses performed in randomized trials, with a focus on target specific anti-cancer drugs and their potential interactions with tumor characteirstics
- Researchers working in duplicate will apply a formal instrument for assessing the credibility of claimed subgroup effects and the extent to which those claims influence recommendations made in subsequent practice guidelines
- A small number of claims could limit the generalizability of our findings
- Another potential limitation could be insufficient reporting of subgroup analyses

Background

The increasing understanding of the biology of malignancies and the availability of new biotechnologies has led to a rapid development of anti-cancer drugs directed at molecular targets. The hope associated with a target-specific (or biomarker-driven) therapy is to maximize anti-cancer effects and minimize side effects. Prominent examples include BRAF inhibitors for melanoma,[1] tyrosine kinase inhibitors for patients with mutated epithelial growth-factor receptor[2] (EGFR), or overexpression of the programmed death ligand 1 protein.[3]

Target-specific anti-cancer drugs are designed to directly inhibit tumor growth (or enhance immunologic anti-tumor response) by influencing a known - or at least partly understood - molecular mechanism. Typically, the targeted mechanism is complex and spans several steps starting with an interaction of the drug with the target molecule, followed by a signaling cascade, leading to endpoints relevant for tumor growth such as proliferation or apoptosis. Alterations of the molecules involved in this mechanism (molecular alterations, MAs) have the potential to modify the effect of the drug.

For peer review only - http://bmjopen.bmj.com/site/about/guidelines.xhtml

Anti-cancer treatments typically have side effects that are judged acceptable under the assumption that the benefits will outweigh the harms. A lack of benefit due to MA could lead to strong effect modifications by altering this balance of benefits and harms of a drug in a way that renders the drug useless or harmful for certain patients.

Investigators of randomized clinical trials (RCTs) increasingly explore effect modifications by MAs in subgroup analyses. A recent survey of cancer trials showed that 103 of 221 (47%) oncology trials published between 2011 and 2013 reported subgroup analyses based on biomarkers.[4]

For instance, an RCT in patients with colorectal cancer addressed the impact of panitumumab, a monoclonal EGFR antibody.[5] The downstream signaling pathway of panitumumab includes proteins encoded by the RAS gene family. Mutations in RAS genes might therefore modify the effect of panitumumab (Figure 1A). A subgroup analysis of that RCT suggested that patients with wildtype RAS proteins have a significant survival benefit (HR 0.78, 95% CI 0.62 – 0.99), whereas patients with a mutation in one of the RAS genes seemed to have worse survival when treated with panitumumab (HR 1.25, 95% CI 1.02 – 1.55, Figure 1B). A statistical test of interaction suggested that chance may not satisfactorily explain the observed subgroup difference (interaction p-value = 0.01).

Another RCT addressed afatinib, which also targets the EGFR pathway (Figure 1C), in patients with lung cancer.[6] The study included patients with a mutation in the EGFR gene, either deletion 19 or substitution Leu858Arg, MAs that might modify the effect of afatinib. Patients with deletion 19 had a significant survival benefit (HR 0.59, 95% CI 0.45 – 0.77), while the effect in patients with substitution Leu858Arg suggested harm (HR 1.25, 95% CI, 0.92 – 1.71, Figure 1D).[6] Chance was an even less likely explanation of the apparent subgroup effect than in the prior example (p=0.0003).

These two examples suggest that target-specific agents may be highly beneficial in one but potentially harmful in another subgroup of patients. The authors of the two trials may have underemphasized the potential risk of giving a target-specific drugs to the inferior subgroup. Instead of acknowledging the potential for serious harm, they concluded a “lack of response”[5] or “absence of effect”[6] in the inferior subgroups. Evidence users may be misled by such conclusions and erroneously assume safety when administering those drugs to patients in whom it may actually cause harm. Moreover, the neglect of possible harm may result in a missed opportunity for further research investigating the reasons and mechanisms for the potential harmful effects.

On the other hand, findings suggested by subgroup analyses have generally low credibility. The majority of claimed subgroup effects are not confirmed in subsequent research[7] and even dramatic looking subgroup effects that are subsequently found to be spurious are not rare.[8,9] There is vast empirical evidence documenting the high risk of spurious subgroup effects.[4,7,10–25] the main reasons being weak hypotheses, multiple testing, selective reporting, and inappropriate statistical analysis.

Some have suggested that subgroup analyses based on MAs, compared to traditional subgroup analyses (e.g. by age or sex), might lead to more credible findings:[26] First, MA-based subgroup effects may have a high biological plausibility (i.e. a clear a priori hypothesis); Second, the side effects typically associated with anti-cancer drugs may –

For peer review only - http://bmjopen.bmj.com/site/about/guidelines.xhtml

in the absence of benefit - actually cause harm, so subgroup effects[27,28] are more likely to be large enough to be detected by statistical tests. For instance, the two examples suggested large qualitative subgroup effects (harmful in one subgroup and beneficial in another) with p-values suggesting chance as unlikely, or very unlikely, explanations.[5,6]

No empirical study is available that addresses MA-based subgroup effects in target-specific anti-cancer therapy. Therefore, we will perform a systematic survey of RCTs to determine if the presented examples are isolated instances or a generalizable phenomenon.

Specific study objectives

1. To assess, in RCTs investigating target-specific anti-cancer drugs, the frequency of
   - subgroup analyses based on MAs
   - claims about subgroup effects made by trial investigators
   - qualitative subgroup effects, i.e. suggesting benefit in one and harm in another subgroup

2. To assess the credibility of claimed subgroup effects based on MAs

3. To assess, for claimed subgroup effects, how trial investigators or guideline developers interpreted the results (e.g. mentioned potential for harm), and how these interpretations relate to the credibility assessment (it would be problematic to emphasize subgroup effects with low credibility or not highlight subgroup effects with high credibility).

For peer review only

For peer review only - http://bmjopen.bmj.com/site/about/guidelines.xhtml

Methods/Design

Sample of randomized trials

We will include publications of parallel-group RCTs that

- enroll patients with a malignant disease;
- include a comparison to investigate the effect of a target-specific anti-cancer drug; and
- report overall or progression free survival (we will focus on survival outcomes only).

We will use an inclusive definition of “target-specific anti-cancer drug” and consider any drug with a defined mode of pharmacodynamic action, e.g. tyrosine kinase inhibitors targeting specific proteins resulting from mutations, all monoclonal antibodies targeting specific proteins, or anti-hormonal drugs (e.g. estrogen or androgen receptor antagonists and/or agonists)

Search strategy

Based on a sensitive search strategy, developed together with a medical information specialist, we have searched MEDLINE via Pubmed for eligible RCTs published since 2014 in the 9 oncology and 4 general medical journals with the highest impact factor, using free text and medical subject headings for cancer and RCTs (Appendix A). Teams of two investigators will screen abstracts for potentially eligible studies, for which we will acquire full texts. Teams of two investigators, one of whom will be an oncologist, will assess final eligibility and, if needed, resolve disagreements by discussion or, if needed, third party adjudication. The teams will also identify reported MA used for subgroup analyses. For RCTs that claim one or more subgroup effects based on MAs, we will screen references and trial registries to identify corresponding protocols, which are relevant for the credibility assessment. For each subgroup claim, we will search corresponding statements in the most current version of clinical guidelines from the American Society of Clinical Oncology (ASCO), European Society of Medical Oncology (ESMO), and UpToDate.com.

Sample size

Our search identified 1119 potentially relevant abstracts of which we already acquired and screened the full texts. Of those, 433 proved eligible RCTs testing a target specific anti-cancer therapy and reporting one or more subgroup analyses. Based on previous research,[4] we estimate that approximately 90 of them will report a subgroup analysis based on a relevant MA. Of those, we estimate that approximately 35 will lead to claimed subgroup effects.[11] This number of RCTs and claims should be sufficient to achieve the study objectives.

For peer review only - http://bmjopen.bmj.com/site/about/guidelines.xhtml

Data extraction

We will develop and standardize extraction forms with detailed explanations for each data point. Teams of two methodologically trained investigators will extract data from publications and associated protocols and resolve disagreements by discussion.

We will extract the following information for each trial: population, interventions, survival outcomes (Overall and progression free survival), the overall treatment effect including confidence intervals, whether the trial reported subgroup analyses in the main paper, appendices, or in secondary publications, number of subgroup hypotheses tested, type of subgroup hypotheses (categorized by oncologists: MA hypothesized to affect effect mechanism, MA outside effect mechanisms such as prognostic biomarkers, other non-molecular prognostic factors, e.g. age or tumor stage, and other variables such as sex, region), subgroup hypotheses (if reported, e.g. a biologic mechanisms explaining the MA might diminish the effect), methods used for subgroup analyses (e.g. interaction term in cox model, forest plot, multivariable analysis of effect modification), results of MA-based subgroup analyses (e.g. point estimates and confidence in each subgroup, p-value from a test of interaction), and whether authors make no, a weak, a moderate, or a strong claim of effect modification (see explanation below).

Assessing strength of subgroup claims

To assess subgroup claims, we will use pre-defined criteria developed by Sun et al. to identify subgroup claims and rate their strength.[11] The criteria address where the authors presented the claim (abstract, conclusion of the abstract, discussion), whether they used descriptive words to strengthen (e.g. using words such as "particular") or soften (e.g. using words such as "might") the claim, obvious notes of caution (e.g. "but not significant"), or indicated that results need to be explored in the future. Based on the assessment, the claims will then be categorized as (1) no claim, (2) suggestion of a possible effect modification, (3) claim of a likely effect modification, (4) or a strong claim of effect modification. Two reviewers will independently assess the presence and strength of claims, collect supporting verbatim quotes, and resolve discrepancies by discussion, if necessary, with the help of a third reviewer.

Assessing credibility of claimed subgroup effects

To assess the credibility of claimed effect modification (for both MAs and other effect modifiers), we will apply a recently developed instrument for assessing the credibility of effect modification analyses (ICEMAN)[30]. In the version for RCTs, the instrument provides 5 core questions:

1. Was the direction of the effect modification correctly hypothesized a priori?
2. Was the effect modification supported by prior evidence?
3. Does a test for interaction suggest that chance is an unlikely explanation of the apparent effect modification?
4. Did the authors test only a small number of effect modifiers or consider the number in their statistical analysis?
5. If the effect modifier is a continuous variable, were arbitrary cut points avoided?

The instrument provides four predefined response for each question and an overall credibility rating on a continuous

For peer review only - http://bmjopen.bmj.com/site/about/guidelines.xhtml

scale ranging from very low to high credibility. Teams of two methodologically trained investigators will independently apply ICEMAN to claimed subgroup effects based on MA and resolve disagreements by discussion. Items 1,2,4, and potentially 5 require a study protocol for optimal assessment. Therefore, for each study reporting a claim, we will search for corresponding study protocols by screening references and trial registry entries.

Statistical analysis

We will use descriptive statistics (frequency, proportions, distribution) to present trial characteristics, including the number and type of subgroup analyses reported per trial, number and strength of claims made, and whether a subgroup claim is qualitative. A qualitative subgroup claims means that a) authors claim a subgroup difference plus b) the point estimates suggest benefit in one harm in another subgroup. (Note that a situation in which one subgroup suggest a hazard ratio of 0.99 and another subgroup a hazard ratio of 1.01 would not count due to lack of claim; authors would make a claim only if the point estimates differ substantially across subgroups).

For all individual subgroup claims, we will present the type of effect modifier (MA versus other), the numerical results, the credibility rating, and the interpretations presented by primary study authors and / or guidelines. We will also create forest plots showing, for all claimed subgroup effects, point estimates and associated confidence intervals within subgroups, differentiating MA-based and other subgroup effects. We will clearly indicate if a trial reports more than one subgroup claim.

A key criterion for the credibility assessment is the result of an interaction test. We know from previous empirical studies, however, that most trials fail to report the results of a test of interaction such as a p-value.[4,7,11] Therefore, whenever possible, we will perform missing interaction tests. For instance, if subgroup specific estimates and confidence intervals are reported, we will calculate the standard errors and approximate the test of interaction by performing a t-test. We will transparently report when we imputed missing data points.

For peer review only

Discussion

This study will provide insights about the effectiveness and safety of target-specific anti-cancer drugs - in particular, in patients subgroups with potentially relevant MAs. Our study will clarify as yet unresolved issues of whether subgroup analyses based on MAs performed as part of RCT reports deliver useful results, and whether trial investigators and guideline developers draw appropriate conclusions.

Several previous studies have systematically assessed the credibility of subgroup effects,[4,7,11] but none has focused on MAs in the context of target-specific anti-cancer drugs. A strength of our study will be the use of a formally developed credibility instrument;[30] previous studies have used ad-hoc criteria or checklists with important limitations.[31] Another strength is that we will focus on subgroup claims that are based on a causal hypotheses and thus likely more credible than subgroup claims based on sex, region, or other characteristics for which there is usually little reason to suspect an effect.[32,33]

We do not anticipate any serious feasibility problems. In previous empirical studies, investigators have successfully used similar methods, including systematic surveys of subgroup analyses in cancer trials,[4] assessing the strength of subgroup claims,[7,11,12] and qualitative analysis of clinical practice guidelines[29]. We anticipate some difficulties in rating the credibility of subgroup claims related to reporting quality. For instance, some trials will not provide a published protocol or insufficiently report the number of effect modifiers tested or numerical results.[18] As implemented in the ICEMAN instrument, we will take the position that insufficient reporting is a reason for lower credibility. Because rating the credibility of subgroup analysis and appropriateness of interpretation inevitably involves judgement, different investigators may come to different conclusions. Therefore, in order to reduce variability of judgements, two investigators will make independent assessments and come to consensus by discussion. In addition, we will provide verbatim quotes to support our judgements in a transparent way.

A limitation of quantitative results such as the frequency of subgroup claims based on MAs in cancer trial will be a high risk of reporting and publication bias. We will therefore make clear that our results will reflect what is reported and not necessarily how often authors consider those analyses. This potential limitation, however, will not weaken inferences made for individual RCTs. Another potential limitation is that, although we know from previous research that subgroup analyses based on biomarkers are included in almost half of oncology trials,[4] we have no specific information about the frequency of MAs-based subgroup analyses. It might be possible that we will identify only a small number of relevant claims based on MA and corresponding guideline statements.

In summary, this systematic survey of RCTs will address characteristics, frequency, credibility, and impact of effect modification analysis based on MAs. If our findings support the alarming hypothesis that the risk of harm caused by target-based drugs might be systematically under-emphasized, our results will help to increase the awareness of the issue among patients, oncologists, trial investigators, and guideline developers. The results may trigger a re-evaluation of existing trials, inform planning of future trials, and influence how trial investigators and guideline developers interpret MA-based subgroup analyses in the context of target-specific anti-cancer therapy.

For peer review only - http://bmjopen.bmj.com/site/about/guidelines.xhtml

Figure legend

Figure 1: (A) Mechanisms of action of panitumumab and molecular alteration of RAS protein. (B) Corresponding randomized clinical trial in patients with colorectal cancer; the trial reports a subgroup analysis for outcome survival suggesting benefit in patients with RAS wildtype tumors but harm in patients with RAS mutated tumors.[5] **(C) Mechanism of action of afatinib and molecular alteration of Epidermal Growth Factor Receptor (EGFR). (D) Corresponding randomized clinical trial in patients with lung cancer; the trial reports a subgroup analysis for outcome survival suggesting benefit in patients with EGFR deletion 19 but harm in patients with EGFR substitution Leu858Arg.**[6]

Declarations

Ethics approval and consent to participate: Not applicable. This study is based on published data only.

Consent for publication: Not applicable

Availability of data and material: We will share the raw data that we extracted from publications.

Competing interests: BK reports consultant activities for Roche and Siemens, research Grants from Roche/AbbVie and travel support from Riemser, AbbVie and Amgen - all not related to the project described herein. All other authors declare that they have no competing interests.

Funding: The study is funded through a personal grant to StS from the Swiss National Science Foundation (project number 180830). The funder had no influence in developing this protocol.

Patients or Public Involvement: It was not appropriate to involve patients or the public in the development of this research proposal.

Authors' contributions: StS, LGH, GHG, MB, and BK conceptualized the study. HE, DG, and BK developed the search strategy and screened titles and abstracts. BK, AH, and AS screened full texts. All authors discussed and edited drafts of the manuscript and read and approved the final version.

Acknowledgements: We thank Kuebra Oezoglu for acquiring the full texts.

For peer review only

1. References

1. McArthur GA, Chapman PB, Robert C, Larkin J, Haanen JB, Dummer R, et al. Safety and efficacy of vemurafenib in BRAF(V600E) and BRAF(V600K) mutation-positive melanoma (BRIM-3): extended follow-up of a phase 3, randomised, open-label study. Lancet Oncol. 2014 Mar;15(3):323–32.

2. Haaland B, Tan PS, de Castro G, Lopes G. Meta-Analysis of First-Line Therapies in Advanced Non–Small-Cell Lung Cancer Harboring EGFR-Activating Mutations. J Thorac Oncol. 2014 Jun 1;9(6):805–11.

3. Reck M, Rodríguez-Abreu D, Robinson AG, Hui R, Csőszi T, Fülöp A, et al. Pembrolizumab versus chemotherapy for PD-L1--positive non--small-cell lung cancer. N Engl J Med. 2016;375(19):1823–33.

4. Zhang S, Liang F, Li W, Hu X. Subgroup Analyses in Reporting of Phase III Clinical Trials in Solid Tumors. J Clin Oncol. 2015 May 20;33(15):1697–702.

For peer review only - http://bmjopen.bmj.com/site/about/guidelines.xhtml

5. Douillard J-Y, Oliner KS, Siena S, Tabernero J, Burkes R, Barugel M, et al. Panitumumab--FOLFOX4 treatment and RAS mutations in colorectal cancer. N Engl J Med. 2013;369(11):1023–34.

6. Yang JC-H, Wu Y-L, Schuler M, Sebastian M, Popat S, Yamamoto N, et al. Afatinib versus cisplatin-based chemotherapy for EGFR mutation-positive lung adenocarcinoma (LUX-Lung 3 and LUX-Lung 6): analysis of overall survival data from two randomised, phase 3 trials. Lancet Oncol. 2015;16(2):141–51.

7. Wallach JD, Sullivan PG, Trepanowski JF, Sainani KL, Steyerberg EW, Ioannidis JPA. Evaluation of Evidence of Statistical Support and Corroboration of Subgroup Claims in Randomized Clinical Trials. JAMA Intern Med. 2017 Apr 1;177(4):554–60.

8. Rothwell PM. Treating individuals 2. Subgroup analysis in randomised controlled trials: importance, indications, and interpretation. Lancet. 2005;365(9454):176–86.

9. Guyatt G. Users' guides to the medical literature : a manual for evidence-based clinical practice. New York: McGraw-Hill Education; 2015.

10. Brookes, ; Whitely ST, ; Egger E, ; Smith M, ; Mulheran GD, ; Peters PA, et al. Subgroup analyses in randomized trials: risks of subgroup-specific analyses; power and sample size for the interaction test. J Clin Epidemiol. 2004;57(3):229–36.

11. Sun X, Briel M, Busse JW, You JJ, Akl EA, Mejza F, et al. Credibility of claims of subgroup effects in randomised controlled trials: systematic review. BMJ. 2012 Mar 15;344:e1553.

12. Saragiotto BT, Maher CG, Moseley AM, Yamato TP, Koes BW, Sun X, et al. A systematic review reveals that the credibility of subgroup claims in low back pain trials was low. J Clin Epidemiol. 2016;79:3–9.

13. Pocock SJ, Assmann SE, Enos LE, Kasten LE. Subgroup analysis, covariate adjustment and baseline comparisons in clinical trial reporting: current practice and problems. Stat Med. 2002;21(19):2917–30.

14. Schuit E, Li AH, Ioannidis JPA. How often can meta-analyses of individual-level data individualize treatment? A meta-epidemiologic study. Int J Epidemiol [Internet]. 2018; Available from: https://www.ncbi.nlm.nih.gov/pubmed/30445577

15. Gabler NB, Duan N, Raneses E, Suttner L, Ciarametaro M, Cooney E, et al. No improvement in the reporting of clinical trial subgroup effects in high-impact general medical journals. Trials. 2016;17(1):320.

16. Donegan S, Williams L, Dias S, Tudur-Smith C, Welton N. Exploring treatment by covariate interactions using subgroup analysis and meta-regression in cochrane reviews: a review of recent practice. PLoS One. 2015;10(6):e0128804.

17. Mistry D, Patel S, Hee SW, Stallard N, Underwood M. Evaluating the quality of subgroup analyses in randomized controlled trials of therapist-delivered interventions for nonspecific low back pain: a systematic review. Spine . 2014;39(7):618–29.

18. Kasenda B, Schandelmaier S, Sun X, von Elm E, You J, Blümle A, et al. Subgroup analyses in randomised controlled trials: cohort study on trial protocols and journal publications. BMJ. 2014 Jul 16;349(jul16 1):g4539.

19. Fernandez YGE, Nguyen H, Duan N, Gabler NB, Kravitz RL. Assessing Heterogeneity of Treatment Effects: Are Authors Misinterpreting Their Results? Health Serv Res. 2010;45(1):283–301.

20. Koopman L, van der Heijden GJ, Hoes AW, Grobbee DE, Rovers MM. Empirical comparison of subgroup effects in conventional and individual patient data meta-analyses. Int J Technol Assess Health Care. 2008;24(3):358–61.

21. Wang R, Lagakos SW, Ware JH, Hunter DJ, Drazen JM. Statistics in medicine--reporting of subgroup analyses in clinical trials. N Engl J Med. 2007;357(21):2189–94.

22. Patsopoulos NA, Tatsioni A, Ioannidis JP. Claims of sex differences: an empirical assessment in genetic associations. JAMA. 2007;298(8):880–93.

23. Hernandez AV, Boersma E, Murray GD, Habbema JD, Steyerberg EW. Subgroup analyses in therapeutic

For peer review only - http://bmjopen.bmj.com/site/about/guidelines.xhtml

cardiovascular clinical trials: are most of them misleading? Am Heart J. 2006;151(2):257–64.

24. Assmann SF, Pocock SJ, Enos LE, Kasten LE. Subgroup analysis and other (mis)uses of baseline data in clinical trials. Lancet. 2000;355(9209):1064–9.

25. Bhandari M, Devereaux PJ, Li P, Mah D, Lim K, Schunemann HJ, et al. Misuse of baseline comparison tests and subgroup analyses in surgical trials. Clin Orthop Relat Res. 2006;447:247–51.

26. Yusuf S, Wittes J. Interpreting Geographic Variations in Results of Randomized, Controlled Trials. N Engl J Med. 2016 Dec 8;375(23):2263–71.

27. Peto R. Statistical aspects of cancer trials. Treatment of cancer. 1982;867–71.

28. Cuzick J. The assessment of subgroups in clinical trials. Experientia Suppl. 1982;41:224–35.

29. Ladanie A, Speich B, Naudet F, Agarwal A, Pereira TV, Sclafani F, et al. The Comparative Effectiveness of Innovative Treatments for Cancer (CEIT-Cancer) project: Rationale and design of the database and the collection of evidence available at approval of novel drugs. Trials. 2018 Sep 19;19(1):505.

30. Stefan Schandelmaier, Matthias Briel, Ravi Varadhan, Christopher Schmid, Phd, Niveditha Devasenapathy, Rodney Hayward, Md, Joel j Gagnier, Michael Borenstein, Geert JMG VanDerHeijden, Issa J Dahabreh, Xin Sun, Willi Sauerbrei, Michael Walsh, John Ioannidis, Lehana Thabane, Gordon Guyatt. A new instrument to assess the credibility of effect modification analyses (ICEMAN) in randomized controlled trials and meta-analyses. Under revision at Annals of Internal Medicine.

31. Schandelmaier S, Chang Y, Devasenapathy N, Devji T, Kwong JS, Colunga Lozano LE, et al. A systematic survey identified 36 criteria for assessing effect modification claims in randomized trials or meta-analyses. J Clin Epidemiol [Internet]. 2019 May 24; Available from: http://dx.doi.org/10.1016/j.jclinepi.2019.05.014

32. Rhodes KM, Turner RM, Higgins JP. Empirical evidence about inconsistency among studies in a pair-wise meta-analysis. Res Synth Methods. 2016;7(4):346–70.

33. Wallach JD, Sullivan PG, Trepanowski JF, Steyerberg EW, Ioannidis JPA. Sex based subgroup differences in randomized controlled trials: empirical evidence from Cochrane meta-analyses. BMJ. 2016 Nov 24;355:i5826.

For peer review only

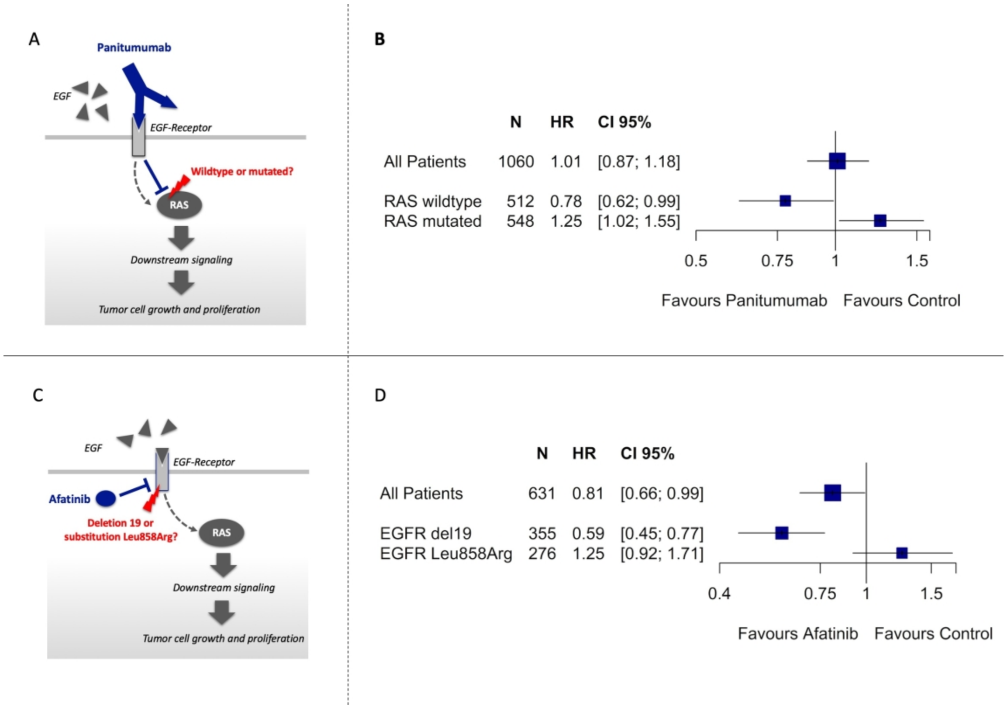

Figure 1: (A) Mechanisms of action of panitumumab and molecular alteration of RAS protein. (B) Corresponding randomized clinical trial in patients with colorectal cancer; the trial reports a subgroup analysis for outcome survival suggesting benefit in patients with RAS wildtype tumors but harm in patients with RAS mutated tumors.[5] (C) Mechanism of action of afatinib and molecular alteration of Epidermal Growth Factor Receptor (EGFR). (D) Corresponding randomized clinical trial in patients with lung cancer; the trial reports a subgroup analysis for outcome survival suggesting benefit in patients with EGFR deletion 19 but harm in patients with EGFR substitution Leu858Arg.[6]

501x350mm (152 x 152 DPI)

MEDLINE/Pubmed search strategy

("Lancet Haematol"[Journal] OR "J Clin Oncol"[Journal] OR "Ann Oncol"[Journal] OR "JAMA Oncol"[Journal] OR "Br J Cancer"[Journal] OR "Lancet Oncol"[Journal] OR "Cancer"[Journal] OR "Blood"[Journal] OR "Leukemia"[Journal]) OR (("N Engl J Med"[Journal] OR "Lancet"[Journal] OR "JAMA"[Journal] OR "Ann Intern Med"[Journal]) AND ("neoplasms"[MeSH Terms] OR cancer [sb] OR neoplas*[tiab] OR cancer*[tiab] OR malignan* OR tumor[tiab] OR tumour[tiab])) AND (randomized controlled trial[pt] OR controlled clinical trial[pt] OR randomized[tiab] OR placebo[tiab] OR "clinical trials as topic"[MeSH Terms:noexp] OR randomly[tiab] OR trial[ti] NOT ("animals"[MeSH Terms] NOT "humans"[MeSH Terms])) AND ("2014/01/01"[PDAT] : "3000/12/31"[PDAT])

For peer review only

PRISMA-P (Preferred Reporting Items for Systematic review and Meta-Analysis Protocols) 2015 checklist: recommended items to address in a systematic review protocol*

Section and topic	Item No	Checklist item	Provided on page
**ADMINISTRATIVE INFORMATION**			
Title:			
Identification	1a	Identify the report as a protocol of a systematic review	1
Update	1b	If the protocol is for an update of a previous systematic review, identify as such	Not applicable
Registration	2	If registered, provide the name of the registry (such as PROSPERO) and registration number	Not registred
Authors:			
Contact	3a	Provide name, institutional affiliation, e-mail address of all protocol authors; provide physical mailing address of corresponding author	1
Contributions	3b	Describe contributions of protocol authors and identify the guarantor of the review	10
Amendments	4	If the protocol represents an amendment of a previously completed or published protocol, identify as such and list changes; otherwise, state plan for documenting important protocol amendments	Not applicable
Support:			
Sources	5a	Indicate sources of financial or other support for the review	10
Sponsor	5b	Provide name for the review funder and/or sponsor	10
Role of sponsor or funder	5c	Describe roles of funder(s), sponsor(s), and/or institution(s), if any, in developing the protocol	10
**INTRODUCTION**			
Rationale	6	Describe the rationale for the review in the context of what is already known	3-5
Objectives	7	Provide an explicit statement of the question(s) the review will address with reference to participants, interventions, comparators, and outcomes (PICO)	5
**METHODS**			
Eligibility criteria	8	Specify the study characteristics (such as PICO, study design, setting, time frame) and report characteristics (such as years considered, language, publication status) to be used as criteria for eligibility for the review	6
Information sources	9	Describe all intended information sources (such as electronic databases, contact with study authors, trial registers or other grey literature sources) with planned dates of coverage	6
Search strategy	10	Present draft of search strategy to be used for at least one electronic database, including planned limits, such that it could be repeated	appendix

For peer review only

Study records:			
Data management	11a	Describe the mechanism(s) that will be used to manage records and data throughout the review	Not provided
Selection process	11b	State the process that will be used for selecting studies (such as two independent reviewers) through each phase of the review (that is, screening, eligibility and inclusion in meta-analysis)	6
Data collection process	11c	Describe planned method of extracting data from reports (such as piloting forms, done independently, in duplicate), any processes for obtaining and confirming data from investigators	7
Data items	12	List and define all variables for which data will be sought (such as PICO items, funding sources), any pre-planned data assumptions and simplifications	7
Outcomes and prioritization	13	List and define all outcomes for which data will be sought, including prioritization of main and additional outcomes, with rationale	Not applicable
Risk of bias in individual studies	14	Describe anticipated methods for assessing risk of bias of individual studies, including whether this will be done at the outcome or study level, or both; state how this information will be used in data synthesis	7
Data synthesis	15a	Describe criteria under which study data will be quantitatively synthesised	Not applicable
	15b	If data are appropriate for quantitative synthesis, describe planned summary measures, methods of handling data and methods of combining data from studies, including any planned exploration of consistency (such as I^2, Kendall's τ)	Not applicable
	15c	Describe any proposed additional analyses (such as sensitivity or subgroup analyses, meta-regression)	Not applicable
	15d	If quantitative synthesis is not appropriate, describe the type of summary planned	Not applicable
Meta-bias(es)	16	Specify any planned assessment of meta-bias(es) (such as publication bias across studies, selective reporting within studies)	Not possible (because those methods require quantitative outcome data)
Confidence in cumulative evidence	17	Describe how the strength of the body of evidence will be assessed (such as GRADE)	Not applicable

*** It is strongly recommended that this checklist be read in conjunction with the PRISMA-P Explanation and Elaboration (cite when available) for important clarification on the items. Amendments to a review protocol should be tracked and dated. The copyright for PRISMA-P (including checklist) is held by the PRISMA-P Group and is distributed under a Creative Commons Attribution Licence 4.0.**

From: Shamseer L, Moher D, Clarke M, Ghersi D, Liberati A, Petticrew M, Shekelle P, Stewart L, PRISMA-P Group. Preferred reporting items for systematic review and meta-analysis protocols (PRISMA-P) 2015: elaboration and explanation. BMJ. 2015 Jan 2;349(jan02 1):g7647.

For peer review only

BMJ Open

Characteristics and interpretation of subgroup analyses based on tumor characteristics in randomized trials testing target-specific anti-cancer drugs: design of a systematic survey

Journal:	*BMJ Open*
Manuscript ID	bmjopen-2019-034565.R1
Article Type:	Protocol
Date Submitted by the Author:	02-Mar-2020
Complete List of Authors:	Schandelmaier, Stefan; University of Basel, Institute for Clinical Epidemiology and Biostatistics, Department of Clinical Research; McMaster University, Health Research Methods, Evidence, and Impact<br>Schmitt, Andreas; University Hospital Basel, Department of Medical Oncology<br>Herbrand, Amanda; University Hospital Basel, Department of Medical Oncology<br>Glinz, Dominik; University of Basel, Institute for Clinical Epidemiology and Biostatistics, Department of Clinical Research<br>Ewald, Hannah; University of Basel, University Medical Library<br>Briel, Matthias; University of Basel, Institute for Clinical Epidemiology and Biostatistics, Department of Clinical Research; McMaster University, Health Research Methods, Evidence, and Impact<br>Guyatt, Gordon; McMaster University, Health Research Methods, Evidence, and Impact; McMaster University, Department of Medicine<br>Hemkens, Lars; University of Basel, Institute for Clinical Epidemiology and Biostatistics, Department of Clinical Research<br>Kasenda, Benjamin; University Hospital Basel, Department of Medical Oncology; iOMEDICO AG, Research and Development
**Primary Subject Heading**:	Oncology
Secondary Subject Heading:	Research methods
Keywords:	Precision medicine, Randomized controlled trials, Subgroup analysis, Systematic survey of studies, ONCOLOGY, STATISTICS & RESEARCH METHODS

SCHOLARONE™
Manuscripts

I, the Submitting Author has the right to grant and does grant on behalf of all authors of the Work (as defined in the below author licence), an exclusive licence and/or a non-exclusive licence for contributions from authors who are: i) UK Crown employees; ii) where BMJ has agreed a CC-BY licence shall apply, and/or iii) in accordance with the terms applicable for US Federal Government officers or employees acting as part of their official duties; on a worldwide, perpetual, irrevocable, royalty-free basis to BMJ Publishing Group Ltd ("BMJ") its licensees and where the relevant Journal is co-owned by BMJ to the co-owners of the Journal, to publish the Work in this journal and any other BMJ products and to exploit all rights, as set out in our licence.

The Submitting Author accepts and understands that any supply made under these terms is made by BMJ to the Submitting Author unless you are acting as an employee on behalf of your employer or a postgraduate student of an affiliated institution which is paying any applicable article publishing charge ("APC") for Open Access articles. Where the Submitting Author wishes to make the Work available on an Open Access basis (and intends to pay the relevant APC), the terms of reuse of such Open Access shall be governed by a Creative Commons licence – details of these licences and which Creative Commons licence will apply to this Work are set out in our licence referred to above.

Other than as permitted in any relevant BMJ Author's Self Archiving Policies, I confirm this Work has not been accepted for publication elsewhere, is not being considered for publication elsewhere and does not duplicate material already published. I confirm all authors consent to publication of this Work and authorise the granting of this licence.

For peer review only

Characteristics and interpretation of subgroup analyses based on tumor characteristics in randomized trials testing target-specific anti-cancer drugs: design of a systematic survey

Authors

Stefan Schandelmaier (1,2), corresponding author, s.schandelmaier@gmail.com

Andreas M Schmitt (3)

Amanda K Herbrand (3)

Dominik Glinz (1)

Hannah Ewald (4)

Matthias Briel (1,2)

Gordon Guyatt (2,5)

Lars G Hemkens (1)

Benjamin Kasenda (3,6)

Affiliations

(1) Institute for Clinical Epidemiology and Biostatistics, Department of Clinical Research, University Hospital and University of Basel, Switzerland
(2) Department of Health Research Methods, Evidence, and Impact, McMaster University, Hamilton, Canada
(3) Department of Medical Oncology, University Hospital Basel, Basel, Switzerland
(4) University Medical Library, University of Basel, Basel, Switzerland
(5) Department of Medicine, McMaster University, Hamilton, Canada
(6) iOMEDICO AG, Research and Development, Freiburg, Germany

For peer review only

Abstract

Background: Target-specific anti-cancer drugs are under rapid development. Little is known, however, about the risk of administering target-specific drugs to patients who have tumors with molecular alterations or other characteristics that make the drug ineffective or even harmful. An increasing number of randomized clinical trials (RCTs) investigating target-specific anti-cancer drugs include subgroup analyses based on tumor characteristics. Such subgroup analyses have the potential to be more credible and influential than subgroup analyses based on traditional factors such as sex or tumor stage. In addition, they may more frequently lead to qualitative subgroup effects, i.e., show benefit in one but harm in another subgroup of patients (e.g. if the tumor characteristicmakes the drug ineffective or even enhance tumor growth). If so, subgroup analyses based on tumor characteristics would be highly relevant for patient safety. To systematically assess the frequency and characteristics of subgroup analyses based on tumor characteristics, the frequency of qualitative subgroup effects, their credibility, and the interpretations that investigators and guidelines developers report.

Methods and analysis: We will perform a systematic survey of 433 RCTs testing the effect of target-specific anti-cancer drugs. Teams of methodologically trained investigators and oncologists will identify eligible studies, extract relevant data, and assess the credibility of putative subgroup effects using a recently developed formal instrument. We will systematically assess how trial investigators interpret apparent subgroup effects based on tumor characteristics and the extent to which they influence subsequent practice guidelines. Our results will provide empirical data characterizing an increasingly used type of subgroup analyses in cancer trials and its potential impact on precision medicine to predict benefit or harm.

Ethics and dissemination: Formal ethical approval is not required for this study. We will disseminate the findings in a peer-reviewed and open-access journal publication.

For peer review only

Strengths and limitations of this study

- We will use rigorous methodology including a systematic search for oncology trials published in leading journals, duplicate data extraction by a team involving both experienced methodologists and oncologists, transparent documentation including the collection of verbatim quotes, and use of a formal instrument for assessing the credibility of claimed subgroup effects.
- The systematic survey will specifically address subgroup claims based on tumor characteristics, which become increasingly relevant for decision making in an era of precision medicine.
- Potential limitations include a small number of eligible subgroup claims based on tumor characteristics, suboptimal reporting of identified subgroup claims and lack of subgroup analysis plans.

For peer review only

Keywords

Oncology
Target-specific anti-cancer treatment
Precision medicine
Randomized controlled trials
Effect modification
Subgroup analysis
Reporting of research
Interpretation of research
Meta-research
Systematic survey of studies

List of abbreviations

BRAF = A proto-oncogene
EGFR = Epithelial Growth Factor Receptor
RAS = Family of proteins relevant for intracellular signaling
RCT = randomized clinical trial

Introduction

The increasing understanding of the biology of malignancies and the availability of new biotechnologies has led to a rapid development of anti-cancer drugs directed at molecular targets. The hope associated with a target-specific (or biomarker-driven) therapy is to maximize anti-cancer effects and minimize side effects. Prominent examples include BRAF inhibitors for melanoma,[1] tyrosine kinase inhibitors for patients with mutated epithelial growth-factor receptor[2] (EGFR), or overexpression of the programmed death ligand 1 protein.[3]

Target-specific anti-cancer drugs are designed to directly inhibit tumor growth, or enhance immunologic anti-tumor response, by influencing a known - or at least partly understood - molecular mechanism. Typically, the targeted mechanism is complex and spans several steps starting with an interaction of the drug with the target molecule, followed by a signaling cascade, leading to endpoints relevant for tumor growth such as proliferation or apoptosis. Alterations of the molecules involved in this mechanism have the potential to modify the effect of the drug.

Anti-cancer treatments typically have side effects that are judged acceptable under the assumption that the benefits will outweigh the harms. A lack of benefit due to relevant alterations could affect this balance of benefits and harms of a drug in a way that renders the drug useless or harmful for certain patients.

Investigators of randomized clinical trials (RCTs) increasingly use subgroup analyses to explore effect modifications by tumor characteristics. Those include subgroup analyses based on specific molecular alterations (e.g. certain BRAF mutations), but also more unspecific tumor characteristics such as measures of mutation burden (i.e. composite variables of several alterations), tumor grade, or histological subtype. A recent survey of cancer trials showed that 103 of 221 (47%) oncology trials published between 2011 and 2013 reported subgroup analyses based on biomarkers.[4]

For peer review only

For instance, an RCT in patients with colorectal cancer addressed the impact of panitumumab, a monoclonal EGFR antibody.[5] The downstream signaling pathway of panitumumab includes proteins encoded by the RAS gene family. Mutations in RAS genes might therefore modify the effect of panitumumab (Figure 1A). A subgroup analysis of that RCT suggested that patients with wildtype RAS proteins have a significant survival benefit (HR 0.78, 95% CI 0.62 – 0.99), whereas patients with a mutation in one of the RAS genes seemed to have worse survival when treated with panitumumab (HR 1.25, 95% CI 1.02 – 1.55, Figure 1B). A statistical test of interaction suggested that chance may not satisfactorily explain the observed subgroup difference (interaction p-value = 0.01).

== Figure 1 ==

Figure 1: (A) Mechanisms of action of panitumumab and molecular alteration of RAS protein. (B) Corresponding randomized clinical trial in patients with colorectal cancer; the trial reports a subgroup analysis for outcome survival suggesting benefit in patients with RAS wildtype tumors but harm in patients with RAS mutated tumors.[5] (C) Mechanism of action of afatinib and molecular alteration of Epidermal Growth Factor Receptor (EGFR). (D) Corresponding randomized clinical trial in patients with lung cancer; the trial reports a subgroup analysis for outcome survival suggesting benefit in patients with EGFR deletion 19 but harm in patients with EGFR substitution Leu858Arg.[6]

Another RCT addressed afatinib, which also targets the EGFR pathway (Figure 1C), in patients with lung cancer.[6] The study included patients with a mutation in the EGFR gene, either deletion 19 or substitution Leu858Arg, genetic alterations that might modify the effect of afatinib. Patients with deletion 19 had a significant survival benefit (HR 0.59, 95% CI 0.45 – 0.77), while the effect in patients with substitution Leu858Arg suggested harm (HR 1.25, 95% CI, 0.92 – 1.71, Figure 1D).[6] Chance was an even less likely explanation of the apparent subgroup effect than in the prior example (p=0.0003).

These two examples suggest that target-specific agents may be highly beneficial in one but potentially harmful in another subgroup of patients. The authors of the two trials may have underemphasized the potential risk of giving a target-specific drugs to the inferior subgroup. Instead of acknowledging the potential for serious harm, they concluded a “lack of response”[5] or “absence of effect”[6] in the inferior subgroups. Evidence users may be misled by such conclusions and erroneously assume safety when administering those drugs to patients in whom it may actually cause harm. Moreover, the neglect of possible harm may result in a missed opportunity for further research investigating the reasons and mechanisms for the potential harmful effects.

On the other hand, findings suggested by subgroup analyses have generally low credibility. The majority of claimed subgroup effects are not confirmed in subsequent research[7] and even dramatic looking subgroup effects that are subsequently found to be spurious are not rare.[8,9] There is vast empirical evidence documenting the high risk of spurious subgroup effects,[4,7,10-25] the main reasons being weak hypotheses, multiple testing, selective reporting, and inappropriate statistical analysis.

For peer review only

Some have suggested that subgroup analyses based on tumor characteristics, compared to traditional subgroup analyses (e.g. by age or sex), might lead to more credible findings:[26] First, MA-based subgroup effects may have a high biological plausibility (i.e. a clear a priori hypothesis); Second, the side effects typically associated with anti-cancer drugs may – in the absence of benefit - actually cause harm and corresponding subgroup effects[2728] may therefore be more likely to be large enough to be detected by statistical tests.27,28 For instance, the two examples suggested large qualitative subgroup effects (harmful in one subgroup and beneficial in another) with p-values suggesting chance as an unlikely, or very unlikely, explanation.[5,6]

No empirical study is available that addresses subgroup effects based on tumor characteristics in target-specific anti-cancer therapy. Therefore, we will perform a systematic survey of RCTs to determine if the presented examples are isolated instances or a generalizable phenomenon.

Specific study objectives are

1) To assess, in RCTs investigating target-specific anti-cancer drugs, the frequency of
    - subgroup analyses based on tumor characteristics
    - claims about subgroup effects based on tumor characteristics made by trial investigators
    - qualitative subgroup effects based on tumor characteristics, i.e. suggesting benefit in one and harm in another subgroup
2) To assess the credibility of claimed subgroup effects based on tumor characteristics
3) To assess, for claimed subgroup effects, how trial investigators or guideline developers interpreted the results (e.g., mentioned potential for harm), and how these interpretations relate to the credibility assessment (e.g., it would be problematic to emphasize subgroup effects with low credibility or not highlight subgroup effects with high credibility).

Methods and analysis

Sample of randomized trials

We will include publications of parallel-group RCTs that (1) enroll patients with a malignant disease; and (2) include a comparison to investigate the effect of a target-specific anti-cancer drug. We will use an inclusive definition of "target-specific anti-cancer drug" and consider any drug with a defined mode of pharmacodynamic action, e.g. tyrosine kinase inhibitors targeting specific proteins resulting from mutations, all monoclonal antibodies targeting specific proteins, or anti-hormonal drugs (e.g. estrogen or androgen receptor antagonists and/or agonists)

Search strategy

Based on a sensitive search strategy that we developed together with a medical information specialist, we searched PubMed for eligible RCTs published since 2014 in the 9 oncology and 4 general medical journals

For peer review only

with the highest impact factor in the year 2016. We used free text and medical subject headings for terms related to cancer and combined them with journal indexations and a validated filter for RCTs.[29] (Appendix A). Teams of two investigators, working independently and in duplicate, will screen abstracts for potentially eligible studies, for which we will acquire full texts. The CEIT cancer database will provide an initial list of target-specific drugs that we will extend during the screening.[30] Teams of two investigators, one of whom will be an oncologist, will assess final eligibility and, if needed, resolve disagreements by discussion or, if needed, third party adjudication. The teams will also independently identify reported tumor characteristics used for subgroup analyses. For RCTs that claim one or more subgroup effects based on tumor characteristics, we will screen references and trial registries to identify corresponding study protocols, which are relevant for the credibility assessment. For each subgroup claim, we will search corresponding statements in the most current version of clinical guidelines from the American Society of Clinical Oncology (ASCO), European Society of Medical Oncology (ESMO), and UpToDate.com.

Sample size

Our search identified 1119 potentially relevant abstracts of which we already acquired and screened the full texts. Of those, 433 proved eligible RCTs testing a target specific anti-cancer therapy and reporting one or more subgroup analyses. Based on previous research,[4] we estimate that approximately 90 of them will report a subgroup analysis based on a tumor characteristic. Of those, we estimate that approximately 35 will lead to claimed subgroup effects.[11] This number of RCTs and claims should be sufficient to achieve the study objectives.

Data extraction

We will develop and standardize extraction forms with detailed explanations for each data point. Teams of two methodologically trained investigators will extract data from publications and associated protocols and resolve disagreements by discussion.

We will extract the following information for each trial: population; interventions; primary outcome(s) , the overall treatment effect including confidence intervals; whether the trial reported any subgroup analyses (or analyses of effect modification based on a continuous variable) in the main paper, appendices, or in secondary publications; number of effect modifiers (i.e. subgrouping variables) type of effect modifiers (categorized by oncologists into (1) tumor characteristics of interest defined as genetic alterations, grade, subtype, or other molecular or histological characteristics; (2) tumor characteristics not of interest such as staging, location, size, or other macroscopic or radiologic characteristics; or (3) other effect modifiers such as age, or sex), subgroup hypotheses (if reported, e.g. a rationale explaining how a genetic alteration might diminish the effect of a target-specific drug), methods used for subgroup analyses (e.g. interaction term in cox model, forest plot, multivariable analysis of effect modification), numerical results of subgroup analyses (e.g. point estimates and confidence in each subgroup, p-value from a test of interaction), and whether authors make no, a weak, a moderate, or a strong claim of effect modification (see explanation below).

For peer review only

Assessing the strength of subgroup claims

To assess the strength of reported subgroup claims, we will use pre-defined criteria developed by Sun et al..[11] The criteria address where the authors presented the claim (abstract, conclusion of the abstract, discussion), whether they used descriptive words to strengthen (e.g. using words such as "particular") or soften (e.g. using words such as "might") the claim, obvious notes of caution (e.g. "but not significant"), or indicated that results need to be explored in the future. Based on the assessment, the claims will then be categorized as (1) no claim; (2) suggestion of a possible effect modification; (3) claim of a likely effect modification; (4) or a strong claim of effect modification. Two reviewers will independently assess the presence and strength of claims, collect supporting verbatim quotes, and resolve discrepancies by discussion, if necessary, with the help of a third reviewer.

Assessing the credibility of claimed subgroup effects

To assess the credibility of claimed effect modification (based on tumor characteristics), we will apply a recently developed instrument for assessing the credibility of effect modification analyses (ICEMAN)[31]. In the version for RCTs, the instrument provides 5 core questions:

1. Was the direction of the effect modification correctly hypothesized a priori?
2. Was the effect modification supported by prior evidence?
3. Does a test for interaction suggest that chance is an unlikely explanation of the apparent effect modification?
4. Did the authors test only a small number of effect modifiers or consider the number in their statistical analysis?
5. If the effect modifier is a continuous variable, were arbitrary cut points avoided?

The instrument provides four predefined response for each question and an overall credibility rating on a continuous scale ranging from very low to high credibility. Teams of two methodologically trained investigators will independently apply ICEMAN to claimed subgroup effects based on tumor characteristics and resolve disagreements by discussion. Items 1,2,4, and potentially 5 require a study protocol for optimal assessment. Therefore, for each study reporting a claim, we will search for corresponding study protocols by screening references and trial registry entries.

Statistical analysis

We will use descriptive statistics (frequency, proportions, distribution) to present trial characteristics, including the number and type of subgroup analyses reported per trial, number and strength of claims made, and whether a subgroup claim is qualitative. A qualitative subgroup claims means that a) authors claim a subgroup difference plus b) the point estimates suggest benefit in one harm in another subgroup. (Note that a situation in which one subgroup suggests a hazard ratio of 0.99 and another subgroup a hazard ratio of 1.01 would not count due to lack of claim. Typically, authors make a claim only if point estimates differ substantially or a test of interaction suggests a p-value of 0.05 or smaller).

For peer review only

For all individual subgroup claims, we will present the type of effect modifier (tumor characteristics versus other), the numerical results, the credibility rating, and the interpretations presented by primary study authors and / or guidelines. We will also create forest plots showing, for all claimed subgroup effects, point estimates and associated confidence intervals within subgroups, differentiating MA-based and other subgroup effects. We will clearly indicate if a trial reports more than one subgroup claim.

A key criterion for the credibility assessment is the result of an interaction test (usually a p-value). We know from previous empirical studies that most trials reporting subgroup effects do not include the results of a test of interaction.[4,7,11] We will report the proportion of studies that report an appropriate test of interaction, present sufficient data to calculate a p-value of interaction, or fail to present a p-value and sufficient data to calculate p-value of interaction. Because here we are interested in the credibility of subgroup claims rather than reporting quality, we will perform missing interaction tests whenever possible. For instance, if subgroup specific estimates and confidence intervals are reported, we will calculate the standard errors and approximate the test of interaction by performing a t-test. We will transparently report when we imputed missing data points.

Patient and Public Involvement

No patients involved.

Discussion

This study will provide insights about the effectiveness and safety of target-specific anti-cancer drugs - in particular, in patients subgroups with potentially relevant tumor characteristics. Our study will clarify as yet unresolved issues of whether subgroup analyses based on tumor characteristics performed as part of RCT reports deliver useful results, and whether trial investigators and guideline developers draw appropriate conclusions.

Several previous studies have systematically assessed the credibility of subgroup effects,[4,7,11] but none has focused on tumor characteristics in the context of target-specific anti-cancer drugs. A strength of our study will be the use of formally developed ICEMAN credibility instrument.[31,32] Previous studies that investigated the credibility of subgroup claims have used ad-hoc criteria or checklists with important limitations including non-transparent development process, criteria based on individual expert opinion rather than consensus, vagueness in criteria, and lack of user-testing.[33] ICEMAN is a new instrument based on a systematic survey of the relevant methodological literature, an explicit measurement concept, a formal instrument development process together with a consensus panel consisting of leading experts, and systematic user-testing.[31,32]Another strength of the study is that we will focus on subgroup claims that are more likely to be based on causal hypotheses and thus likely more credible than subgroup claims based on sex, region, or other characteristics for which there is usually little reason to suspect effect modification.[34,35]

For peer review only

We do not anticipate any serious feasibility problems. In previous empirical studies, investigators have successfully used similar methods, including systematic surveys of subgroup analyses in cancer trials,[4] assessing the strength of subgroup claims,[7,11,12] and qualitative analysis of clinical practice guidelines.[30] We anticipate some difficulties in rating the credibility of subgroup claims related to reporting quality. For instance, some trials will not provide a published protocol or insufficiently report the number of effect modifiers tested or numerical results.[18] As implemented in the ICEMAN instrument, we will take the position that insufficient reporting is a reason for lower credibility. Because rating the credibility of subgroup analysis and appropriateness of interpretation inevitably involves judgement, different investigators may come to different conclusions. Therefore, in order to reduce variability of judgements, two investigators will make independent assessments and come to consensus by discussion. In addition, we will provide verbatim quotes to support our judgements in a transparent way.

A limitation of quantitative results such as the frequency of subgroup claims based on tumor characteristics in cancer trial will be a high risk of reporting and publication bias. We will therefore make clear that our results will reflect what is reported and not necessarily how often authors consider those analyses. This potential limitation, however, will not weaken inferences made for individual RCTs. Another potential limitation is that, although we know from previous research that subgroup analyses based on biomarkers are included in almost half of oncology trials,[4] we have no specific information about the frequency of subgroup analyses based on tumor characteristics. It might be possible that we will identify only a small number of relevant claims based on tumor characteristics and corresponding guideline statements.

In summary, this systematic survey of RCTs will address characteristics, frequency, credibility, and impact of effect modification analysis based on tumor characteristics. If our findings support the alarming hypothesis that the risk of harm caused by target-based drugs might be systematically under-emphasized, our results will help to increase the awareness of the issue among patients, oncologists, trial investigators, and guideline developers. The results may trigger a re-evaluation of existing trials, inform planning of future trials, and influence how trial investigators and guideline developers interpret MA-based subgroup analyses in the context of target-specific anti-cancer therapy.

Ethics and dissemination

Formal ethical approval is not required for this study that will be based on published information only. We will disseminate the findings in a peer-reviewed and open-access journal publication

Full references

1. McArthur GA, Chapman PB, Robert C, Larkin J, Haanen JB, Dummer R, et al. Safety and efficacy of vemurafenib in BRAF(V600E) and BRAF(V600K) mutation-positive melanoma (BRIM-3): extended follow-up of a phase 3, randomised, open-label study. Lancet Oncol. 2014 Mar;15(3):323–32.

For peer review only

2. Haaland B, Tan PS, de Castro G, Lopes G. Meta-Analysis of First-Line Therapies in Advanced Non-Small-Cell Lung Cancer Harboring EGFR-Activating Mutations. J Thorac Oncol. 2014 Jun 1;9(6):805-11.

3. Reck M, Rodríguez-Abreu D, Robinson AG, Hui R, Csőszi T, Fülöp A, et al. Pembrolizumab versus chemotherapy for PD-L1--positive non--small-cell lung cancer. N Engl J Med. 2016;375(19):1823-33.

4. Zhang S, Liang F, Li W, Hu X. Subgroup Analyses in Reporting of Phase III Clinical Trials in Solid Tumors. J Clin Oncol. 2015 May 20;33(15):1697-702.

5. Douillard J-Y, Oliner KS, Siena S, Tabernero J, Burkes R, Barugel M, et al. Panitumumab--FOLFOX4 treatment and RAS mutations in colorectal cancer. N Engl J Med. 2013;369(11):1023-34.

6. Yang JC-H, Wu Y-L, Schuler M, Sebastian M, Popat S, Yamamoto N, et al. Afatinib versus cisplatin-based chemotherapy for EGFR mutation-positive lung adenocarcinoma (LUX-Lung 3 and LUX-Lung 6): analysis of overall survival data from two randomised, phase 3 trials. Lancet Oncol. 2015;16(2):141-51.

7. Wallach JD, Sullivan PG, Trepanowski JF, Sainani KL, Steyerberg EW, Ioannidis JPA. Evaluation of Evidence of Statistical Support and Corroboration of Subgroup Claims in Randomized Clinical Trials. JAMA Intern Med. 2017 Apr 1;177(4):554-60.

8. Rothwell PM. Treating individuals 2. Subgroup analysis in randomised controlled trials: importance, indications, and interpretation. Lancet. 2005;365(9454):176-86.

9. Guyatt G. Users' guides to the medical literature : a manual for evidence-based clinical practice. New York: McGraw-Hill Education; 2015.

10. Brookes, ; Whitely ST, ; Egger E, ; Smith M, ; Mulheran GD, ; Peters PA, et al. Subgroup analyses in randomized trials: risks of subgroup-specific analyses; power and sample size for the interaction test. J Clin Epidemiol. 2004;57(3):229-36.

11. Sun X, Briel M, Busse JW, You JJ, Akl EA, Mejza F, et al. Credibility of claims of subgroup effects in randomised controlled trials: systematic review. BMJ. 2012 Mar 15;344:e1553.

12. Saragiotto BT, Maher CG, Moseley AM, Yamato TP, Koes BW, Sun X, et al. A systematic review reveals that the credibility of subgroup claims in low back pain trials was low. J Clin Epidemiol. 2016;79:3-9.

13. Pocock SJ, Assmann SE, Enos LE, Kasten LE. Subgroup analysis, covariate adjustment and baseline comparisons in clinical trial reporting: current practice and problems. Stat Med. 2002;21(19):2917-30.

14. Schuit E, Li AH, Ioannidis JPA. How often can meta-analyses of individual-level data individualize treatment? A meta-epidemiologic study. Int J Epidemiol [Internet]. 2018; Available from: https://www.ncbi.nlm.nih.gov/pubmed/30445577

15. Gabler NB, Duan N, Raneses E, Suttner L, Ciarametaro M, Cooney E, et al. No improvement in the reporting of clinical trial subgroup effects in high-impact general medical journals. Trials. 2016;17(1):320.

16. Donegan S, Williams L, Dias S, Tudur-Smith C, Welton N. Exploring treatment by covariate interactions using subgroup analysis and meta-regression in cochrane reviews: a review of recent practice. PLoS One. 2015;10(6):e0128804.

17. Mistry D, Patel S, Hee SW, Stallard N, Underwood M. Evaluating the quality of subgroup analyses in randomized controlled trials of therapist-delivered interventions for nonspecific low back pain: a systematic review. Spine . 2014;39(7):618-29.

18. Kasenda B, Schandelmaier S, Sun X, von Elm E, You J, Blümle A, et al. Subgroup analyses in randomised controlled trials: cohort study on trial protocols and journal publications. BMJ. 2014 Jul 16;349(jul16 1):g4539.

For peer review only

19. Fernandez YGE, Nguyen H, Duan N, Gabler NB, Kravitz RL. Assessing Heterogeneity of Treatment Effects: Are Authors Misinterpreting Their Results? Health Serv Res. 2010;45(1):283-301.

20. Koopman L, van der Heijden GJ, Hoes AW, Grobbee DE, Rovers MM. Empirical comparison of subgroup effects in conventional and individual patient data meta-analyses. Int J Technol Assess Health Care. 2008;24(3):358-61.

21. Wang R, Lagakos SW, Ware JH, Hunter DJ, Drazen JM. Statistics in medicine--reporting of subgroup analyses in clinical trials. N Engl J Med. 2007;357(21):2189-94.

22. Patsopoulos NA, Tatsioni A, Ioannidis JP. Claims of sex differences: an empirical assessment in genetic associations. JAMA. 2007;298(8):880–93.

23. Hernandez AV, Boersma E, Murray GD, Habbema JD, Steyerberg EW. Subgroup analyses in therapeutic cardiovascular clinical trials: are most of them misleading? Am Heart J. 2006;151(2):257-64.

24. Assmann SF, Pocock SJ, Enos LE, Kasten LE. Subgroup analysis and other (mis)uses of baseline data in clinical trials. Lancet. 2000;355(9209):1064-9.

25. Bhandari M, Devereaux PJ, Li P, Mah D, Lim K, Schunemann HJ, et al. Misuse of baseline comparison tests and subgroup analyses in surgical trials. Clin Orthop Relat Res. 2006;447:247-51.

26. Yusuf S, Wittes J. Interpreting Geographic Variations in Results of Randomized, Controlled Trials. N Engl J Med. 2016 Dec 8;375(23):2263-71.

27. Peto R. Statistical aspects of cancer trials. Treatment of cancer. 1982;867–71.

28. Cuzick J. The assessment of subgroups in clinical trials. Experientia Suppl. 1982;41:224–35.

29. Lefebvre C, Manheimer E, Glanville J. Searching for studies. In: Higgins J, Green S, editors. Cochrane Handbook for Systematic Reviews of Interventions Version 510 (updated March 2011).

30. Ladanie A, Speich B, Naudet F, Agarwal A, Pereira TV, Sclafani F, et al. The Comparative Effectiveness of Innovative Treatments for Cancer (CEIT-Cancer) project: Rationale and design of the database and the collection of evidence available at approval of novel drugs. Trials. 2018 Sep 19;19(1):505.

31. Stefan Schandelmaier, Matthias Briel, Ravi Varadhan, Christopher Schmid, Phd, Niveditha Devasenapathy, Rodney Hayward, Md, Joel j Gagnier, Michael Borenstein, Geert JMG VanDerHeijden, Issa J Dahabreh, Xin Sun, Willi Sauerbrei, Michael Walsh, John Ioannidis, Lehana Thabane, Gordon Guyatt. A new instrument to assess the credibility of effect modification analyses (ICEMAN) in randomized controlled trials and meta-analyses. Under revision in CMAJ.

32. Schandelmaier S. Evaluating the credibility of effect modification claims in randomized controlled trials and meta-analyses. Doctoral thesis published on macsphere [Internet]. 2019; Available from: http://hdl.handle.net/11375/24375

33. Schandelmaier S, Chang Y, Devasenapathy N, Devji T, Kwong JS, Colunga Lozano LE, et al. A systematic survey identified 36 criteria for assessing effect modification claims in randomized trials or meta-analyses. J Clin Epidemiol [Internet]. 2019 May 24; Available from: http://dx.doi.org/10.1016/j.jclinepi.2019.05.014

34. Rhodes KM, Turner RM, Higgins JP. Empirical evidence about inconsistency among studies in a pair-wise meta-analysis. Res Synth Methods. 2016;7(4):346-70.

35. Wallach JD, Sullivan PG, Trepanowski JF, Steyerberg EW, Ioannidis JPA. Sex based subgroup differences in randomized controlled trials: empirical evidence from Cochrane meta-analyses. BMJ. 2016 Nov 24;355:i5826.

For peer review only

Authors' contributions

StS, LGH, GHG, MB, and BK conceptualized the study. HE, DG, and BK developed the search strategy and screened titles and abstracts. BK, AH, and AS screened full texts. All authors discussed and edited drafts of the manuscript and read and approved the final version.

Acknowledgements

We thank Kuebra Oezoglu for acquiring the full texts.

Funding statement

The work was supported by a personal grant to StS from the Swiss National Science Foundation (project number 180830).

Competing interests statement

BK reports consultant activities for Roche and Siemens, research Grants from Roche/AbbVie and travel support from Riemser, AbbVie and Amgen - all not related to the project described herein. All other authors declare that they have no competing interests.

Word count: 2942

For peer review only

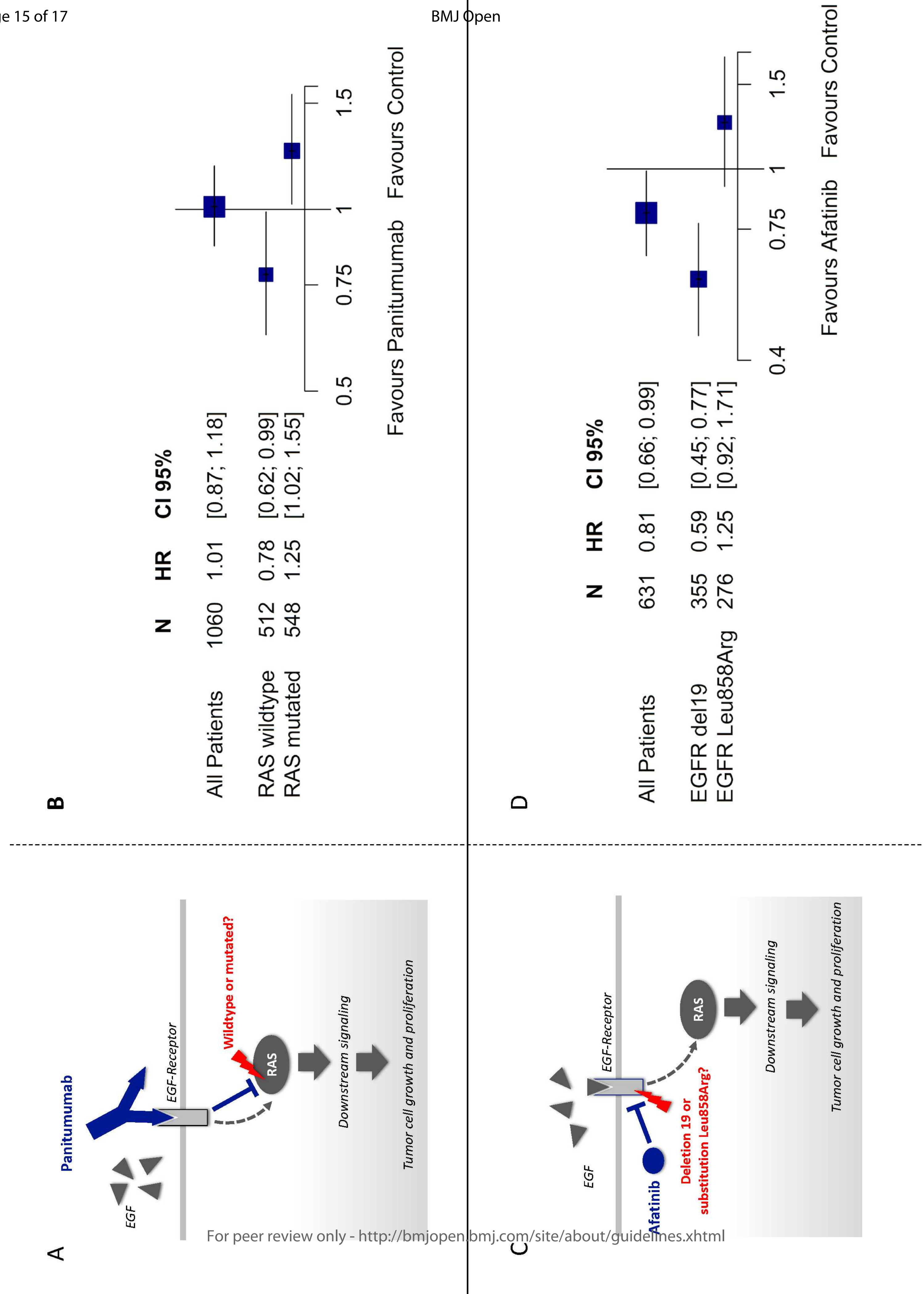

For peer review only - http://bmjopen.bmj.com/site/about/guidelines.xhtml

Supplement

PubMed search strategy:

("Lancet Haematol"[Journal] OR "J Clin Oncol"[Journal] OR "Ann Oncol"[Journal] OR "JAMA Oncol"[Journal] OR "Br J Cancer"[Journal] OR "Lancet Oncol"[Journal] OR "Cancer"[Journal] OR "Blood"[Journal] OR "Leukemia"[Journal]) OR (("N Engl J Med"[Journal] OR "Lancet"[Journal] OR "JAMA"[Journal] OR "Ann Intern Med"[Journal]) AND ("neoplasms"[MeSH Terms] OR cancer [sb] OR neoplas*[tiab] OR cancer*[tiab] OR malignan* OR tumor[tiab] OR tumour[tiab])) AND (randomized controlled trial[pt] OR controlled clinical trial[pt] OR randomized[tiab] OR placebo[tiab] OR "clinical trials as topic"[MeSH Terms:noexp] OR randomly[tiab] OR trial[ti] NOT ("animals"[MeSH Terms] NOT "humans"[MeSH Terms])) AND ("2014/01/01"[PDAT] : "3000/12/31"[PDAT])

Last date of search 20/09/2017; number of identified articles: 7729

For peer review only

PRISMA-P (Preferred Reporting Items for Systematic review and Meta-Analysis Protocols) 2015 checklist: recommended items to address in a systematic review protocol*

Section and topic	Item No	Checklist item	Provided on page
**ADMINISTRATIVE INFORMATION**			
Title:			
Identification	1a	Identify the report as a protocol of a systematic review	1
Update	1b	If the protocol is for an update of a previous systematic review, identify as such	Not applicable
Registration	2	If registered, provide the name of the registry (such as PROSPERO) and registration number	Not registred
Authors:			
Contact	3a	Provide name, institutional affiliation, e-mail address of all protocol authors; provide physical mailing address of corresponding author	1
Contributions	3b	Describe contributions of protocol authors and identify the guarantor of the review	10
Amendments	4	If the protocol represents an amendment of a previously completed or published protocol, identify as such and list changes; otherwise, state plan for documenting important protocol amendments	Not applicable
Support:			
Sources	5a	Indicate sources of financial or other support for the review	10
Sponsor	5b	Provide name for the review funder and/or sponsor	10
Role of sponsor or funder	5c	Describe roles of funder(s), sponsor(s), and/or institution(s), if any, in developing the protocol	10
**INTRODUCTION**			
Rationale	6	Describe the rationale for the review in the context of what is already known	3-5
Objectives	7	Provide an explicit statement of the question(s) the review will address with reference to participants, interventions, comparators, and outcomes (PICO)	5
**METHODS**			
Eligibility criteria	8	Specify the study characteristics (such as PICO, study design, setting, time frame) and report characteristics (such as years considered, language, publication status) to be used as criteria for eligibility for the review	6
Information sources	9	Describe all intended information sources (such as electronic databases, contact with study authors, trial registers or other grey literature sources) with planned dates of coverage	6
Search strategy	10	Present draft of search strategy to be used for at least one electronic database, including planned limits, such that it could be repeated	appendix

For peer review only

Study records:			
Data management	11a	Describe the mechanism(s) that will be used to manage records and data throughout the review	Not provided
Selection process	11b	State the process that will be used for selecting studies (such as two independent reviewers) through each phase of the review (that is, screening, eligibility and inclusion in meta-analysis)	6
Data collection process	11c	Describe planned method of extracting data from reports (such as piloting forms, done independently, in duplicate), any processes for obtaining and confirming data from investigators	7
Data items	12	List and define all variables for which data will be sought (such as PICO items, funding sources), any pre-planned data assumptions and simplifications	7
Outcomes and prioritization	13	List and define all outcomes for which data will be sought, including prioritization of main and additional outcomes, with rationale	Not applicable
Risk of bias in individual studies	14	Describe anticipated methods for assessing risk of bias of individual studies, including whether this will be done at the outcome or study level, or both; state how this information will be used in data synthesis	7
Data synthesis	15a	Describe criteria under which study data will be quantitatively synthesised	Not applicable
	15b	If data are appropriate for quantitative synthesis, describe planned summary measures, methods of handling data and methods of combining data from studies, including any planned exploration of consistency (such as I^2, Kendall's τ)	Not applicable
	15c	Describe any proposed additional analyses (such as sensitivity or subgroup analyses, meta-regression)	Not applicable
	15d	If quantitative synthesis is not appropriate, describe the type of summary planned	Not applicable
Meta-bias(es)	16	Specify any planned assessment of meta-bias(es) (such as publication bias across studies, selective reporting within studies)	Not possible (because those methods require quantitative outcome data)
Confidence in cumulative evidence	17	Describe how the strength of the body of evidence will be assessed (such as GRADE)	Not applicable

*** It is strongly recommended that this checklist be read in conjunction with the PRISMA-P Explanation and Elaboration (cite when available) for important clarification on the items. Amendments to a review protocol should be tracked and dated. The copyright for PRISMA-P (including checklist) is held by the PRISMA-P Group and is distributed under a Creative Commons Attribution Licence 4.0.**

From: Shamseer L, Moher D, Clarke M, Ghersi D, Liberati A, Petticrew M, Shekelle P, Stewart L, PRISMA-P Group. Preferred reporting items for systematic review and meta-analysis protocols (PRISMA-P) 2015: elaboration and explanation. BMJ. 2015 Jan 2;349(jan02 1):g7647.

For peer review only
